# Management of neonates with respiratory distress syndrome in resource-limited settings

**DOI:** 10.4102/safp.v66i1.5938

**Published:** 2024-05-22

**Authors:** Radhika Singh, Leann P. Munian, Nqobile A. Memela

**Affiliations:** 1 Department of Paediatrics, Faculty of Health Sciences, University of KwaZulu-Natal, Durban, South Africa; 2 Department of Biomedical and Clinical Technology, Faculty of Health Sciences, Durban University of Technology, Durban, South Africa

**Keywords:** CPAP, RDS, prematurity, surfactant, low- and middle-income countries, non-invasive respiratory support

## Abstract

In South Africa, prematurity stands as one of the foremost causes of neonatal mortality. A significant proportion of these deaths occur because of respiratory distress syndrome of prematurity. The implementation of non-invasive respiratory support, such as continuous positive airway pressure (CPAP), has demonstrated both safety and efficacy in reducing mortality rates and decreasing the need for mechanical ventilation. Given the absence of blood gas analysers and limited radiological services in many district hospitals, the severity of respiratory distress is often assessed through observation of the infant’s work of breathing and the utilisation of bedside scoring systems. Based on the work of breathing, non-invasive therapy can be commenced timeously. While evidence supporting the use of high-flow nasal cannula as a primary treatment for respiratory distress syndrome remains limited, it may be considered as an alternative, provided that CPAP machines are available. The purpose of this article is to advocate the use of non-invasive therapy in low resource-limited settings and describe the indications, contraindications, complications, and application of CPAP therapy. This would benefit healthcare workers, especially in low-care settings and district hospitals.

## Introduction

With advances in neonatal care, the survival rate of infants less than 1500 g has increased. However, there is a disparity in neonatal outcomes between high-income and low- and middle-income countries. Among the greater than 2.6 million neonatal deaths worldwide each year, more than 98% occur in low- and middle-income countries.^1^ Among the 8 sustainable development goal (SDG) regions, sub-Saharan Africa had the highest neonatal mortality rate in 2018 at 28 deaths per 1000 live births, followed by Central and Southern Asia with 25 deaths per 1000 live births.^2^ In South Africa, the most common cause of death in the neonatal period is prematurity (35% – 48%), followed by intrapartum-related events.^3^

## Neonatal respiratory distress syndrome

Primary respiratory distress syndrome (RDS) from surfactant deficiency in the newborn is the most prevalent respiratory disorder among preterm infants. Respiratory distress syndrome-specific mortality in low- and middle-income countries is high. Survival among neonates less than 32 weeks of gestation is below 50% in resource-limited settings;^4^ however, this has increased over the years to around 70% because of increased access to health care. Improving access to facilities capable of delivering quality neonatal care for small and sick newborns has been identified as a target of the Early Neonatal Action Plan (ENAP), which aims to have 80% of districts with available care for small and sick newborns.^5^ Respiratory distress syndrome is treatable with continuous positive airway pressure (CPAP).

## Continuous positive airway pressure

Continuous positive airway pressure has been used extensively since the 1970s for managing respiratory disease in premature infants and is no longer limited to regional and tertiary hospitals. It is now advocated and widely implemented in district hospitals. It is considered the initial mode of respiratory support for spontaneously breathing preterm infants and is being increasingly used in low- and middle-income countries. It is a relatively low-cost therapy shown to improve neonatal and infant outcomes across a wide range of healthcare settings.^6^ Early use of CPAP in neonates with RDS reduces mortality, the need for mechanical ventilation (MV) and transfer to a tertiary centre.^7,8^ Continuous positive airway pressure has shown a 50% reduction in the need for MV.^9^ Bubble CPAP is cost effective and can be used in district hospitals for neonates with RDS. Continuous positive airway pressure improves lung volume, especially functional residual capacity. The increased positive distending airway pressure improves oxygenation, decreases apnoea and reduces the work of breathing with a reduction in the need for MV and mortality.^10,11^ However, there is an increase in the rate of pneumothorax with CPAP.^11^

## Assessment of a neonate with respiratory distress syndrome in resource-limited settings

One of the challenges in district hospitals (a hospital that receives referrals from and provides generalist support to clinics and community health centres with health treatment administered by general health care practitioners or primary health care nurses) is the lack of early radiological diagnosis and blood gas analysis. There is now less emphasis on radiographic diagnosis and grading of RDS, such as ‘ground glass with air bronchograms’. Definitions based on blood gas analyses are also redundant, as management has moved towards an approach of pre-emptive treatment with surfactant based on clinical assessment of work of breathing and inspired oxygen requirement to avoid worsening RDS.

**FIGURE 1:**
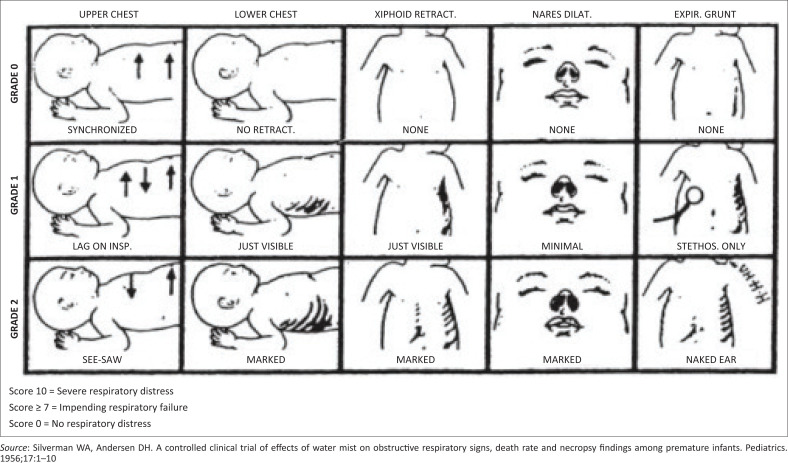
Silverman scoring system.

Work of breathing can be determined using a scoring system that is simple, non-invasive, inexpensive and has shown both prognostic value and good inter-rater reliability. Some of the scoring systems include the Downes scoring system^12^ (Table 1) or the Silverman Andersen respiratory severity scoring system^13^ (Figure 1).

**TABLE 1:** Downes scoring system.

Test	0	1	2
Respiratory rate	< 60	60–80	> 80
-Retractions	No retractions	Mild retractions	Severe retractions
Cyanosis	No cyanosis	Cyanosis relieved by O_2_	Cyanosis on O_2_
Air entry	Good bilateral air entry	Mild decrease in air entry	No air entry
Grunting	No grunting	Audible with stethoscope	Audible with ear

*Source*: Downes JJ, Vidyasagar D, Boggs TR, Jr, Morrow 3rd, GM. Respiratory distress syndrome of newborn infants. I. New clinical scoring system (RDS score) with acid-base and blood-gas correlations. Clin Pediatr. 1970;9(6):325–331. https://doi.org/10.1177/000992287000900607

Note: Score: < 4 = no/mild respiratory distress; 4–7 = respiratory distress; > 7 = impending respiratory failure.

Silverman Andersen score in the range of 4–6 is commonly used as a threshold for initiation, and titration of CPAP therapy given it can be serially repeated at the bedside as a type of vital sign. A score of ≥ 6 on the Downes score can be used to initiate CPAP. Surfactant therapy using the intubate, surfactant and extubate technique (INSURE) can be administered, based on the expertise of the healthcare worker.^14^ Less invasive surfactant administration (LISA) has also proven to be effective; however, more studies are required.^15^ A mechanical ventilator should be available with these techniques, in the event of the neonate failing extubation. Surfactant therapy should be considered if the fraction of inspired oxygen (FiO_2_) is more than 30% and if CPAP pressure of ≥6 cm of water is required. The oxygen target should be between 90% and 94%.

### Continuous positive airway pressure delivery

According to the European consensus guidelines, spontaneously breathing preterm infants should be stabilised using CPAP in the delivery room, with a CPAP pressure of at least 6 cm H_2_O, and peak inspiratory pressures of 20 cm H_2_O – 25 cm H_2_O.^10^ A T-piece device can be used in the delivery room, to deliver these pressures. Continuous positive airway pressure should be continued in the neonatal unit with CPAP pressure between 5 cm and 9 cm of water for spontaneously breathing preterm neonates with RDS.

Methods of CPAP delivery include the following^16^:

- Bubble CPAP – It generates positive pressure by submersion of the expiratory limb of the CPAP circuit under water. The depth of the expiratory limb underwater determines the airway-distending pressure delivered.
- Variable flow CPAP – It generates a positive pressure at the airway, proximal to the neonate’s nose. Bilevel CPAP/DuoPAP can be used to deliver the flow.
- High-flow nasal cannula (HFNC) – It utilises a compressed gas source or flow generator to provide air and oxygen at flow rates of 2 L/min – 8 L/min, with weaning of flow rate determined clinically by FiO_2_ remaining low and a judgement of work of breathing. More studies are required before it is recommended as the first line of management of RDS.

In summary, the system delivering CPAP is of little importance; however, the interface should be short binasal prongs or masks with a starting pressure of about 6 cm H_2_O – 8 cm H_2_O. Bilevel nasal positive airway pressure (BiPAP) does not offer more advantages over bubble CPAP. Synchronised non-invasive positive pressure ventilation (NIPPV), if delivered through a ventilator, can reduce the need for ventilation or the need for re-ventilation following extubation. High-flow nasal cannula can be used as an initial mode of support provided there is access to CPAP.^10^

## Weaning of a neonate on continuous positive airway pressure

There is a lack of consensus regarding the optimal method of weaning neonate off CPAP. Criteria used in various studies to wean a neonate off CPAP are described in Box 1.^17^ This can be determined by the work of breathing. It is variable in different centres.

BOX 1 Stability criteria for weaning of nasal continuous positive airway pressure.

**The following should be present for 24–48 h before weaning:**

CPAP pressure of 5 cm H_2_O;

FiO_2_ of 0.21;

Normal work of breathing: no persistent tachypnoea (> 60 breaths for > 2 h), marked retractions;

No apnoea (cessation of respiration > 20 s) associated with bradycardia or cyanosis with > 2 episodes in 12 h or > 3 in 24 h with at least one requiring bag and mask ventilation;

Saturation > 90% most of the time or PaO_2_/transcutaneous PaO_2_ > 45 mmHg;

Not currently treated for PDA or sepsis at the time of weaning;

Tolerated time off CPAP during nursing cluster care for up to 15 min or more.

*Source:* Reynolds P, Bustani P, Darby C, et al. Less-invasive surfactant administration for neonatal respiratory distress syndrome: A consensus guideline. Neonatology. 2021;118(5):586–592. https://doi.org/10.1159/000518396

CPAP, continuous positive airway pressure; PDA, patent ductus arteriosus; FiO_2_, fractional inspired oxygen; PaO_2_, partial pressure of arterial oxygen.

## Criteria to define continuous positive airway pressure failure

The criteria that can be used to determine the failure of CPAP are as stated in Box 2.^18^

BOX 2 Criteria for failed continuous positive airway pressure (at least two of the following).

- Increase work of breathing (intercostal recession and accessory muscles contributing to respiration) with respiratory rate > 75
- Increased apnoea and/or bradycardia and/or desaturations > 2 in 1 h for the previous 6-hour period, on CPAP
- Increasing oxygen requirement > 25% to maintain the oxygen saturations > 86% and/or PaO_2_ > 45 mmHg
- pH of < 7.2
- PaCO_2_/transcutaneous PaCO_2_ > 65 mmHg
- Major apnoea or bradycardia requiring resuscitation

*Source*: Adapted from Todd DA, Wright A, Broom M, et al. Methods of weaning preterm babies < 30 weeks gestation off CPAP: A multicenter randomised controlled trial. Archiv Dis Childhood Fetal Neonatal Ed. 2012;97(4):F236–F240. https://doi.org/10.1136/adc.2011-300133-

CPAP, continuous positive airway pressure

## Indications, contra-indications and complications of nasal continuous positive airway pressure in preterm infants

### Indications

Continuous positive airway pressure is primarily used in premature babies who are breathing spontaneously but require more support than nasal prong oxygen, typically those requiring more than 40% FiO_2_.^19,20^ Physical signs warranting its use include severe respiratory distress, apnoea, cyanosis and/or grunting. In hospitals with access to a blood gas machine, respiratory acidosis may also prompt its utilisation. Continuous positive airway pressure may be utilised in conjunction with surfactant, depending on its availability and the skill set of the attending healthcare providers.

Other indications include term neonates with respiratory distress because of transient tachypnoea of the newborn, congenital pneumonia or meconium aspiration syndrome, once hypoglycaemia and hypothermia have been excluded.^21,22,23^ In the context of a neonatal intensive care unit, CPAP may be used in patients being weaned off a conventional ventilator or high-frequency oscillation. Furthermore, in resource-limited settings, it may be a useful adjunct in patients who do not qualify for MV, such as infants weighing between 800 g and 1000 g.^24^

### Contra-indications

Nasal CPAP is contra-indicated in various congenital and surgical disorders, including but not limited to^21^:

- Upper Airway: Cleft palate, Bilateral choanal atresia.
- Lower Airway: Trachea-oesophageal atresia, Congenital diaphragmatic hernia, Suspected or confirmed necrotising enterocolitis.
- Abdominal: Gastroschisis, Congenital airway/lung lesions, post-abdominal surgery.

### Complications

Complications may arise from the equipment, nursing care or the infant’s clinical condition itself:^25,26,27,28^

- Nasal and facial trauma is the most common and well-described complication. Risk factors described are lower gestational age and birth weight, duration of CPAP, lack of use of a protective hydro-colloid barrier, incorrect mask or nasal prong size and healthcare provider expertise. These complications can be mitigated by the use of a daily CPAP checklist or by inspecting the nasal septum and nares at least once during a 12-hour shift. This also allows suctioning and cleaning as necessary.
- Abdominal distension, also known as CPAP belly, is more common in micropreemies and may delay gastric emptying with a resultant delay in establishing full enteral feeds. An orogastric tube should be passed and aspirated for air between feeds.
- Pulmonary air leak syndromes, including pneumothorax, may occur because of airway distending pressure. Although a rare complication, patients need to be monitored for sudden increases in respiration, desaturation, decreased air entry, and asymmetrical chest rise.
- Infants in a neonatal intensive care unit are prone to hearing loss because of environmental noise and the use of ototoxic medications such as aminoglycosides and diuretics. A CPAP machine adds to this environmental noise. Audiology screening is, therefore, recommended in this high-risk cohort of patients.
- Heat and chemical burns of the skin and mucus membranes are rare complications associated with malfunctioning equipment. Flow rates should be maintained between 6 L/min and 8 L/min, and circuits should be regularly checked.
- Swallowing or aspiration of nasal interface components are additional rare adverse events and may occur if small pieces at the nasal interface are loosely connected.
- There may be a delay in intubation. The use of CPAP might give the healthcare provider a false sense of security; therefore, regular clinical review of the patient with a blood gas, if available, is essential to ensure that CPAP is a successful intervention.

### When to refer patients on continuous positive airway pressure

Consider further intervention if any of the following are present^21^:

- Respiratory distress worsens – clinical signs include increasing tachypnoea, recession, cyanosis and/or grunting.
- Poor respiratory drive – periods of hypoventilation or recurrent apnoeas despite caffeine administration.
- Persistent increase in FiO_2_ requirement – any infant requiring more than 30% FiO_2_ after a few hours, with an increased work of breathing.
- Worsening respiratory acidosis – evidenced by a rise in PCO_2_ and a lower pH.
- Hemodynamic instability – hypotension and poor perfusion requiring fluid resuscitation and/or inotropic support.

Regular consultation with and advice from a referral centre is recommended.

## Set-up of a continuous positive airway pressure machine

Ensuring the proper setup and utilisation of nasal CPAP (nCPAP) is contingent upon various requirements. One needs to emphasise delivering safe and effective respiratory support to neonates.

- *Requirements for nasal CPAP (nCPAP)* continuous positive airway pressure machine:
  ■ The fundamental requirement is a specialised CPAP machine designed exclusively for neonatal use.
  ■ These machines are designed to administer a continuous flow of air and oxygen, effectively maintaining an open upper airway.^29^
  ■ It is imperative to ensure that there is a reliable power source or a backup power supply for uninterrupted operation.^30^
- Supply of medical air and oxygen:
  ■ A dependable source of medical air and oxygen is necessary for connecting to the nCPAP machine.^29^
  ■ In the absence of a centralised air and oxygen system, an alternative involves utilising an oxygen cylinder and an air compressor.^27,28^
  ■ Notably, compatibility between the air compressor and the nCPAP machine is crucial, and the oxygen cylinder may require frequent replacement.
  ■ The recommended gas flow rate for generating CPAP typically ranges from 5 L/min to 10 L/min.^29^
- Generator set with nasal interface:
  ■ The nasal interface, which can be prongs or a mask (Duke),^29^ serves to connect the infant’s airway with the nCPAP circuit.
  ■ Nasal prongs, similar to standard oxygen prongs, are particularly suitable for use in developing countries.^29^
- Hat/headgear:
  ■ A hat or headgear is essential to secure and position the flow driver and nasal interface on the infant’s nose.
- Patient circuit with humidifying chamber:
  ■ An appropriate patient circuit, including a humidifying chamber, is vital for delivering gases from the nCPAP machine to the infant.
  ■ Duke et al. emphasise the inclusion of a heated humidifier in the nCPAP machine, utilising a chamber filled with sterile water to introduce moisture into the administered air, preventing the drying of the infant’s airways.
- Trained healthcare professionals:
  ■ Trained healthcare professionals are indispensable for the setup, operation and monitoring of nCPAP to ensure its safe and effective use.
  ■ Proper training is essential for the healthcare staff involved in administering nCPAP to neonates, emphasising the significance of their role in providing optimal respiratory support.^30^

## Application for nasal continuous positive airway pressure

- Patient assessment: Before initiating nCPAP, it is crucial to conduct a thorough patient assessment. Ensure that the infant is adequately warm with a normal blood glucose level. Following prescribed neonatal guidelines for managing respiratory distress, healthcare professionals can determine the most suitable respiratory support option.
- Proper sizing of hat/head gear and nasal interface: Using a measuring tape, healthcare professionals assess the infant’s head size to select a hat or headgear of the correct size that fits snugly. It is essential to avoid choosing headgear that is either too large, hindering the fit of the flow driver and compromising delivered pressure, or too small, risking potential oedema and discomfort for the baby. The selection of an appropriate nasal interface, such as nasal prongs or nasal masks, using the manufacturer’s guide is vital for effectively delivering pressurised air to the infant’s airways.
  ■ Setting the pressure: Prescribed pressure settings are determined based on the infant’s respiratory needs, typically by healthcare professionals.
  ■ Monitoring and adjustment: Continuous monitoring of the infant’s respiratory status is crucial. Observations include work of breathing, frequency of apnoea, oxygenation, air entry and chest movement. Vital signs are also closely monitored at the bedside.^27^ Pressure levels may be adjusted based on the infant’s response to maintain optimal respiratory support.
  ■ Patient care: Thukal et al.^31^ and Dada et al.^30^ state that the holistic utilisation and application of (nCPAP in neonatal care are essential for ensuring its safe and effective implementation within district hospitals. It includes user-friendly equipment, readily available and correct utilisation of consumables, optimal pressure settings, continuous monitoring, ongoing training and attentive patient care.^30^ Maintaining a secure fit of the hat/headgear, flow driver and nasal interface is essential, and healthcare professionals must avoid overly tight fittings. Regular pressure-relieving interventions on the nose, ears, forehead, nasal bridge and head are performed to prevent pressure injuries and skin breakdown. Thirty years of adopting this comprehensive approach, district hospitals can ensure the successful and beneficial use of nCPAP, ultimately leading to positive outcomes for infants grappling with respiratory distress.

## Feasibility of nasal continuous positive airway pressure

Evaluating the feasibility of implementing nCPAP in district hospitals is essential for successful integration, sustained operation and positive health outcomes for patients in need of respiratory support. In resource-limited settings, diagnostic tests such as laboratory and radiographic assessments are not consistently available. This is compounded by the lack of medical doctor supervision, leading nurses to take the initiative in medical interventions.^30,32^ Initiating nCPAP is an easy process; however, for optimal effectiveness, it requires continuous usage for hours or days, involving a consistent supply of electricity and medical gases, along with ongoing clinical monitoring.^33^ Nasal continuous positive airway pressure stands out as a promising and reliable technology for delivering respiratory support to neonates facing respiratory distress.^29,31^ Its potential for extensive adoption, especially in addressing respiratory distress in developing nations, is emphasised by its lower initial costs. Challenges such as the cost and availability of consumables, the need for additional equipment like humidifiers and the presence of skilled staff may hinder the widespread implementation of nCPAP therapy.^31^

## The feasibility assessment of nasal continuous positive airway pressure in district hospitals encompasses the following

- Financial considerations: To introduce nCPAP services, a thorough assessment of financial feasibility is imperative. This involves scrutinising the costs associated with equipment purchase, procurement of consumables, staff training and system maintenance.^29^ Identifying potential funding sources and understanding budgetary implications will be crucial for sustainable implementation.
- Infrastructure: The existing infrastructure of district hospitals must be examined to ascertain its capacity to support the installation and operation of nCPAP machines. This includes evaluating the availability of medical gases and electrical capacity to ensure uninterrupted functionality.^30^ Continuous positive airway pressure, if provided using air or minimal additional oxygen titrated to safe oxygen saturation, can help mitigate what has been described as a second epidemic of retinopathy of prematurity in developing countries.
- Resource availability: Evaluating essential resources such as consumables, monitors and humidifiers is critical for the optimal operation of nCPAP.^30^ Ensuring a steady supply of these resources for maintaining continuous and effective respiratory support, is crucial.
- Training and education: The proficiency of healthcare professionals in utilising nCPAP, troubleshooting issues and managing patients is fundamental. Robust training programmes should be implemented to enhance the skills and knowledge of healthcare staff. Regular training sessions are essential to ensure optimal nCPAP delivery and contribute to successful long-term implementation.

By addressing these key aspects, healthcare facilities can pave the way for the successful implementation of nCPAP, ultimately improving respiratory support for infants in resource-limited settings.

## Conclusion

Respiratory distress syndrome stands out as a significant contributor to neonatal mortality and morbidity. Utilising non-invasive CPAP emerges as a safe and efficient approach in mitigating both mortality and morbidity among preterm neonates. Its applicability within district hospitals proves advantageous, as it minimises the necessity for neonatal transfers to regional and tertiary care centres, thereby alleviating strain on the healthcare system. Nonetheless, substantial training is imperative before implementing CPAP at lower levels of care.
